# An immune-related prognostic model predicts neoplasm-immunity interactions for metastatic nasopharyngeal carcinoma

**DOI:** 10.3389/fimmu.2023.1109503

**Published:** 2023-03-31

**Authors:** Xiaochuan Chen, Qin Ding, Ting Lin, Yingming Sun, Zongwei Huang, Ying Li, Wenquan Hong, Xin Chen, Desheng Wang, Sufang Qiu

**Affiliations:** ^1^ Department of Radiation Oncology, Clinical Oncology School of Fujian Medical University, Fujian Cancer Hospital, Fuzhou, China; ^2^ Fujian Provincial Key Laboratory of Translational Cancer Medicine, Clinical Oncology School of Fujian Medical University, Fujian Cancer Hospital, Fuzhou, China; ^3^ Department of Radiation and Medical Oncology, Affiliated Sanming First Hospital of Fujian Medical University, Sanming, China; ^4^ Department of Otolaryngology, Fujian Medical University Union Hospital, Fuzhou, China

**Keywords:** immunotherapy, bioinformatics, metastatic nasopharyngeal carcinoma, immune microenvironment, mRNA transcriptome sequencing and single cell sequencing

## Abstract

**Background:**

The prognosis of nasopharyngeal carcinoma (NPC) has been recognized to improve immensely owing to radiotherapy combined with chemotherapy. However, patients with metastatic NPC have a poor prognosis. Immunotherapy has dramatically prolonged the survival of patients with NPC. Hence, further research on immune-related biomarkers is imperative to establish the prognosis of metastatic NPC.

**Methods:**

10 NPC RNA expression profiles were generated from patients with or without distant metastasis after chemoradiotherapy from the Fujian Cancer Hospital. The differential immune-related genes were identified and validated by immunohistochemistry analysis. The method of least absolute shrinkage and selection operator (LASSO)was used to further establish the immune-related prognostic model in an external GEO database (GSE102349, n=88). The immune microenvironment and signal pathways were evaluated in multiple dimensions at the transcriptome and single-cell levels.

**Results:**

1328 differential genes were identified, out of which 520 were upregulated and 808 were downregulated. Notably, most of the immune genes and pathways were down-regulated in the metastasis group. A prognostic immune model involving nine hub genes. Patients in low-risk group were characterized by survival advantage, hot immune phenotype and benefit from immunotherapy. Compared with immune cells, malignant cell exhibited the most active levels of risk score by ssGSEA. Accordingly, intercellular communications including LT, CD70, CD40 and SPP1, and the like, between high-risk and low-risk were explored by the R package “Cellchat”.

**Conclusion:**

We have constructed a model based on immunity of metastatic NPC and determined its prognostic value. The model identified the level of immune cell infiltration, cell-cell communication, along with potential immunotherapy for metastatic NPC.

## Introduction

Nasopharyngeal carcinoma (NPC), an Epstein-Barr virus (EBV)-associated cancer that is prevalent in Southern China (1), has been recognized to have a favorable prognosis owing to radiotherapy combined with chemotherapy during the past decades. Apart from EBV infection, human papillomavirus (HPV) infection, alcohol and tobacco consumption, smoking, and the consumption of salt-preserved foods have recently been identified as high-risk factors (2). Although most patients reach complete clinical remission, it has been suggested that patients with recurrence or metastasis have a poor prognosis. The application of intensity-modulated radiotherapy has improved the treatment outcome of NPC, especially the local control rate, but the impact on distant metastasis is minimal. The 5-year survival rate of patients with early-stage NPC can reach more than 90% with a relatively low rate of 60% for patients with advanced stage (3). Hence, currently, the focus should be on the cure of metastatic NPC. Exploring new therapeutic targets and developing new molecularly targeted drugs are definitely the direction of future research. In addition, exploring the molecular mechanism of distant metastasis of NPC and screening high-risk groups will also facilitate individualized response in the initial treatment.

Immune checkpoint blockade (ICB)-based immunotherapy, such as programmed cell death ligand 1 (PD-L1) and interferon (IFN)-γ, has dramatically changed the treatments of cancer to prolong the patients’ survival (4). Particularly, the clinical research of immunotherapy has contributed majorly to the individual treatments of malignant tumors (5). However, the most well-known research recommends Pembrolizumab as the first-line treatment for PD-L1-positive recurrent or metastatic head and neck squamous cell carcinomas (6). The response to immunotherapy for the treatment of metastatic NPC is inconclusive.

In recent years, the assessment of immunotherapy efficacy has become a major challenge for clinicians to individualize treatment. Although no accepted immune-related risk model for predicting prognosis exists, the reported models have shown decent predictive validity in certain cancers (7–12). The focus of immune-related prediction models is not only restricted to the genomic level but also extended to the transcriptome level, single-cell level, and so on (13–18). However, there are not many studies on immune-related prognosis models integrating single-cell RNA and mRNA levels in metastatic NPC. Therefore, it is of great significance to explore novel immune-related diagnostics and therapeutics for patients with metastatic NPC.

This study aimed to (i) identify the immune-related genes, (ii) reveal the underlying pathway associated with metastatic NPC, (iii) establish the prognostic immune model and evaluate its prognostic value, and (iv) validate the predictive validity of the model from various aspects.

## Materials and methods

### Patients’ samples

10 NPC tumor tissue samples were obtained from the patients who were diagnosed and treated at the Fujian Cancer Hospital between May 9, 2013, and August 2, 2016. All 10 patients met the following eligibility criteria: newly diagnosed NPC, received standardized radiotherapy and chemotherapy, ≥18 years old, adequate hematological, renal and hepatic functions, and no other malignant diseases. All the patients provided written informed consent. The study was approved by the Ethics Committee of Fujian Cancer Hospital and Fujian Medical University Cancer Hospital (approval number SQ2019-035-01). The tissue samples were stored in liquid nitrogen for subsequent RNA extraction. During the 5 years of follow-up, 5 samples were from patients with disease progression after radiotherapy and chemotherapy, containing 3 liver metastases, 1 bone metastasis, and 1 lung metastasis. While the other 5 samples were evaluated as having a complete or partial response after the treatment.

As an external validation cohort, RNA-seq data of NPC from the GEO database (https://www.ncbi.nlm.nih.gov/geo/, GSE102349) were selected to verify the reliability and applicability of the data of this study (19, 20). The single-cell dataset GSE150430 was designed to validate the accuracy of the model at the individual cell level and to probe the communication of cells and ligand receptors in the immune microenvironment of NPC. Also, the tumor tissue biopsies of 74 NPC patients treated in our hospital in 2021 and 2022 were used for the immunohistochemistry to validate CD8 T cell infiltration and immune checkpoints expression, including 11 cases in the metastatic group and 63 cases in the non-metastatic group (**Supplementary Table 1**). The 8th edition of the American Joint Committee on Cancer (AJCC) Staging Manual was used to restage all the patients.

### Immunohistochemistry analysis

NPC biopsies were fixed with 10% formalin overnight and processed into 5-μm-thick paraffin sections. The slides were then analyzed by immunohistochemistry with anti-human CD8 (Cat # ab237709; Abcam), anti-human PD1 (Cat # ab52587; Abcam), and anti-human PD-L1 (Cat # ab213524; Abcam) followed by HRP secondary antibody (Cat #ab205718; Abcam) and DAB staining. Images were obtained using a microscope (BX43; Olympus, Japan). Histochemistry score (H-score) was used to evaluate the expression. H-score = (percentage of cells of weak intensity × 1) + (percentage of cells of moderate intensity × 2) + (percentage of cells of strong intensity × 3).

### Construction and validation of immune-related prognostic model

The R package “ggplot2” was employed to visualize DEGs from sequencing data of NPC samples in Fujian Cancer Hospital (21). The cut-off values met the following two conditions: fold-change of >2 and the p-value of<0.05. Gene ontology (GO) (22, 23) and Kyoto Encyclopedia of Genes and Genomes (KEGG) pathway analyses (24) were applied to further explore the pathways of DEGs enrichment. A false-discovery rate of<0.05 was set as the cut-off value. The immune gene data was downloaded through the ImmPort data portal (www.immport.org/immport-open/public/home/home), and 2,498 immune-related genes were obtained. Then the intersection of the DEGs and the immune-related genes was selected as differentially expressed immune-related genes. Progression-free survival (PFS) was subjected to minimum absolute shrinkage and selection operator (LASSO) Cox regression with 10-fold cross-validation to screen for DEGs with prognostic value on the basis of the univariate Cox analysis. The R package “glmnet” was employed to determine the gene signatures containing the biomarkers most helpful for prognosis (25). The prognosis risk score was established by linearly combining the following formula:


$$\text{risk score}=\sum_{1}^{\text{n}}\left(\text{exp}\times \text{coef}\right)$$

where exp denotes the gene expression value, while coef refers to the coefficient of a gene in LASSO analysis.

To assess the predictive power of our prognostic risk model, receiver operating characteristic (ROC) for 1- and 3-year survival were performed in the validation cohort GSE102349 using the R package “timeROC”. Next, the samples were divided into high-risk and low-risk groups according to the best cut-off value of the risk score from the R package “survival” for survival analysis. The survival curves were compared using the Kaplan-Meier method and the log-rank test. The univariate and multivariate Cox regression models were applied to determine whether the risk score was an independent prognostic factor.

### Immune- and carcinogenesis-related estimation in multiple dimensions

To evaluate the infiltration of immune cells from several aspects, we adopted multiple immune scoring approaches, like TIMER and ssGSEA algorithms (26, 27). The immune scores and tumor purity were estimated by the R package “ESTIMATE” (28). From an earlier study, we retrieved a group of six inhibitory immune checkpoints that displayed immune therapeutic efficacy (29). Gene sets that displayed T cell-inflamed gene expression profile (GEP) and tertiary lymphatic structure (TLS) were acquired (30, 31). Furthermore, we assessed the enrichment of 10 oncogenic pathways using the ssGSEA method (32). The score of activation minus the score of repression represented the final score of each pathway. We used a validated set of 31 genes related to cell cycle progression (CCP) to estimate the rate of cell proliferation (33). The cluster score was calculated as the average expression level of CCP-related pathways by subtracting the mean level.

### Prediction of the immunotherapy response

To assess the predictive efficacy of the model for immunotherapy efficacy, we collected several immunotherapy cohorts from the GEO database and the TIGER website (http://tiger.canceromics.org/#/), including nasopharyngeal carcinoma-GSE102349, melanoma-GSE91061, melanoma-PRJEB23709, NSCLC-GSE126044. We visually compared the proportion of patients with and without response to immunotherapy in high- and low-risk groups.

### Single-cell RNA-seq analysis

This study performed quality control, downscaling, and clustering of scRNA-seq data as well using Seurat (v.4.0.4) (34). To ensure data quality, genes detected in less than 3 cells and cells with less than 250 genes detected were excluded, and the percentage of mitochondria was limited to less than 35% (35). Data were processed by the logNormalize method for normalization. The nonlinear dimensionality reduction method Uniform Manifold Approximation and Projection for Dimension Reduction (UMAP) was utilized for unsupervised classification and unbiased visualization of cell populations on two-dimensional maps (36). TISCH (http://tisch.comp-genomics.org/) provides detailed cell type annotations at the single-cell level (35). After that, the “FindAllMarkers” function was configured to identify marker genes in each cluster using a filter value of absolute log2 fold change (FC) ≥ 0.3 and a minimum cell cluster fraction of 0.25.

### Risk score calculation in single-cell samples

A risk score of each single cell sample from GSE150430 was calculated by single sample Gene Set Enrichment Analysis (ssGSEA) method and was completed using the “GSVA” and “GSEABase” packages in R. We used single-cell data as a reference, apply a newly developed deconvolution algorithms (CIBERSORTx) to the bulk transcriptome data to quantitatively estimate cell-type proportions for each tumor in GEO database (37).

### Cell–cell chat analysis

CellChat v1.1.3 software inferred cell-cell communication based on ligand-receptor interactions (38). Cell groups with less than 10 cells were filtered out of cell-cell communication. Pairwise tests were performed on communication probability values to assess their statistical significance.

### Statistical analysis

Statistical analysis was done using R software (V.3.6.1) and SPSS software (ver. 25.0). Wilcoxon rank sum test and chi-square test were conducted for continuous and categorical variables, respectively. For all analysis, two-by-two pairs indicate statistically significant differences. *, **, *** and **** indicate, respectively <0.05, <0.01, <0.001, and <0.0001.

## Results

### Identification of differential immune-related expressions in NPC

**Figure 1:**
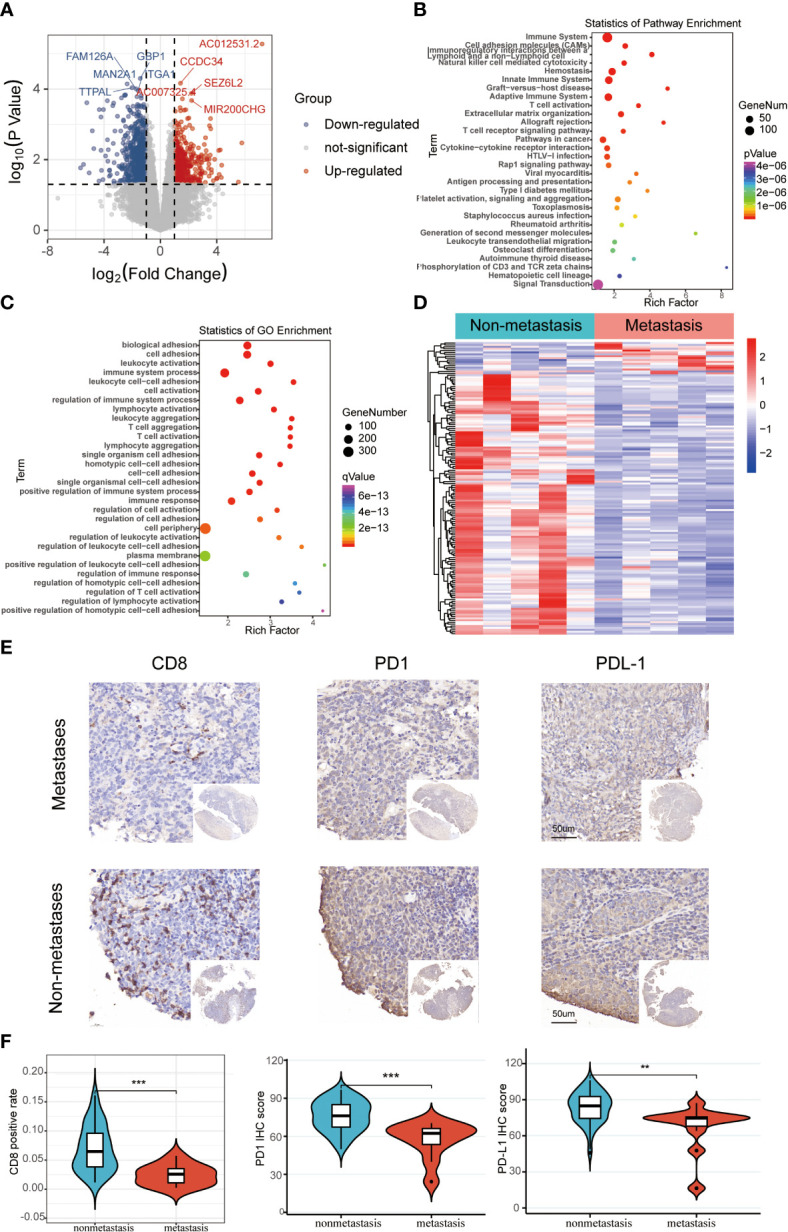
The differentially expressed immune-related genomic biomarkers in nasopharyngeal carcinoma (NPC). **(A)** All 1328 differential genes assessed from the tumor tissues are shown in the volcano plot; red dots for upregulated genes (520 genes), while blue dots represent downregulated genes (808 genes); **(B, C)** Statistics of enrichment analysis using KEGG and GO were concentrated on the immune-related cellular components, biological processes and pathways in the bubble charts; **(D)** The top 154 immune-related genes were significantly differentiated between the metastasis and non-metastasis group in the heatmap; **(E, F)** Immunohistochemical staining results of CD8, PD1, and PD-L1 in metastatic and non-metastatic NPC samples from Fujian Cancer Hospital. **P < 0.01, ***P < 0.001.

The schematic diagram presents the workflow of our study (**Figure S1**). The RNA-seq profiles were generated for the NPC samples of 10 patients treated at the Fujian Cancer Hospital, 5 of whom were assigned to the non-metastasis group, while the other 5 were in the metastasis group owing to distant metastasis after chemoradiotherapy. The baseline characteristics of patients in the metastatic and non-metastatic groups could be seen in **Table 1** (n=10). In general, PCA indicated distinct transcriptional profiles between the metastatic group and the non-metastatic group (**Figure S2A**). Then, 1328 DEGs were conspicuously illustrated in the volcano plot, with 520 upregulated genes and 808 downregulated genes (**Figure 1A**). The KEGG and GO analyses are the universally applicable statistical methods of enrichment analysis. The DEGs were enriched in the immune-related pathways of the bubble chart containing signal transduction, adaptive immune system, innate immune system, and hemostasis (**Figure 1B**). Simultaneously, they were also centralized in the cell periphery, plasma membrane, and immune system processes (**Figure 1C**). The expression levels of the top 154 immune-related genes selected from the DEGs can be significantly distinguished between the two groups in the heat-map (**Figure 1D**). Overall, immune gene expression and immune signaling pathway were down-regulated in the metastasis group, indicating a potential “immune-cold” tumor phenotype in the metastasis group. For the validation, Therefore, we performed immunohistochemistry staining of CD8 T cell, PD1, and PD-L1. Our immunohistochemistry analysis showed that PD1 and PD-L1 expressions were down-regulated, and the infiltration of CD8 T cells was decreased in the metastasis NPC group (n=11) compared to the non-metastasis group (n=63, **Figures 1E, F**).

**Table 1:** The baseline characteristics of patients in the metastatic and non-metastatic groups (n=10).

Variables	metastatic group (n=5)	non-metastatic group (n=5)	*P* value^a^
Gender			*1.000*
Male	*4*	*4*	
Female	*1*	*1*	
Age			*0.167*
≤5*0*	*2*	*5*	
>5*0*	*3*	*0*	
T stage			*0.524*
T1-*2*	*1*	*3*	
T3-*4*	*4*	*2*	
N stage			*1.000*
N0-*1*	*1*	*2*	
N2-*3*	*4*	*3*	
M stage			*0.008*
M*0*	*0*	*5*	
M*1*	*5*	*0*	
Clinical stage			*0.008*
II-III	*0*	*5*	
IV	*5*	*0*	
Survival			*0.048*
Alive	*1*	*5*	
Dead	*4*	*0*	
Pathological type ^b^			*1.000*
WHO I	*0*	*1*	
WHO II	*1*	*1*	
WHO III	*4*	*3*	

a *P* values were two-sided using Fisher’s exact test, ^b^Pathological type includes WHO type I: keratinizing squamous cell carcinoma, WHO type II: non-keratinizing differentiated carcinoma and WHO type III: non-keratinizing undifferentiated carcinoma.

### Establishment and validation of the risk model

**Figure 2:**
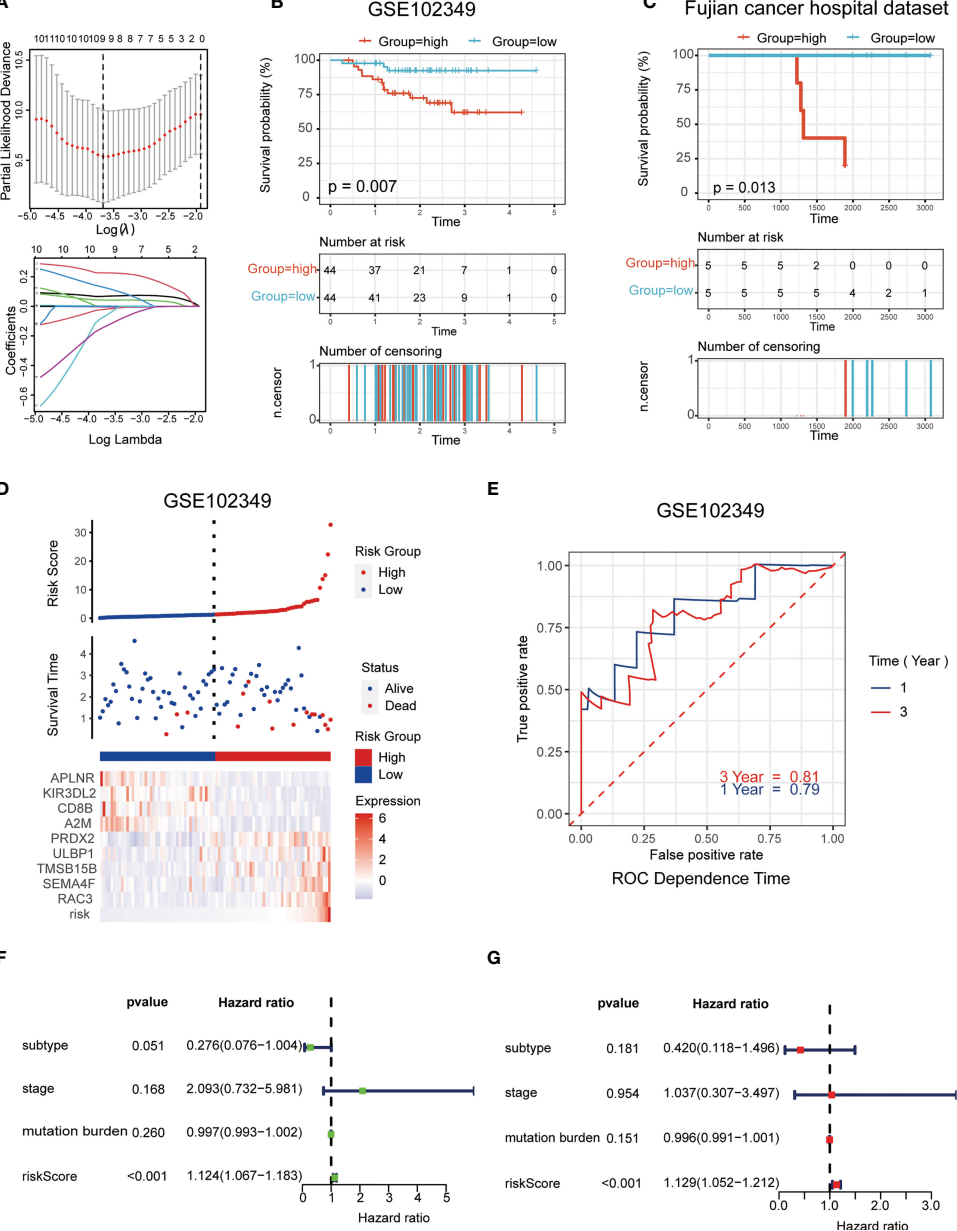
Establishment and validation of the immune-related risk model. **(A)** The LASSO logistic regression model was applied to establish prognostic immune biomarkers which involved 9 signatures (A2M, APLNR, CD8B, RAC3, PRDX2, ULBP1, TMSB15B, KIR3DL2 and SEMA4F) identified by the GEO dataset (GSE102349); **(B, C)** The Kaplan-Meier plot of the immune-related genes in GSE102349 **(B)** and Fujian Cancer Hospital corhort **(C)** revealed the statistical significance between the high- and low-risk groups; **(D)** Patient survival status and expression of 9 hub genes in high and low risk groups; **(E)** Receiver operating characteristic (ROC) curves of 1-year and 3-year survival in GSE102349; **(F, G)** Univariate **(F)** and multivariate **(G)** Cox regression analyses for the immune-related risk score model as an independent prognostic factor.

The LASSO logistic regression model was applied to establish the prognostic immune biomarkers, which involved 9 hub genes (A2M, APLNR, CD8B, RAC3, PRDX2, ULBP1, TMSB15B, KIR3DL2, and SEMA4F; **Figure 2A**). The standard for high and low risk scores was evaluated based on cut points associated with the median risk score. Cut-off value of 1.31 for the risk model was identified, which served to divide the patients into high-risk group (with levels of risk score ≥ 1.31) and a low-risk group (with levels of risk score< 1.31). The risk scores were significantly distinguished between the clinical stages I–III and stage IV in GSE102349 (**Figure S2B**), which indicated that the clinical stage of the tumor could be one of the critical factors in assessing the effect of the treatment. The risk scores were also apparently different between the metastasis and non-metastasis groups in our hospital cohort (**Figure S2C**). Patients in the high-risk group had worse tumor metastatic presentation, which is indicative of a worse prognosis (**Figure 2B**). This finding was further validated in a cohort of patients from Fujian Cancer Hospital (**Figure 2C**).

It was found that APLNR, KIR3DL2, CD8B, and A2M were upregulated in the low-risk group, while PRDX2, ULBP1, TMSB15B, SEMA4F and RAC3 were upregulated in the high-risk group (**Figure 2D**). The assumption could be proposed that the former 4 genes were protective biomarkers, while the latter 5 genes were risk biomarkers. The area under the ROC curve (AUC) was 0.79 at 1-year, and 0.81 at 3-years, respectively, indicating a high predictive value (**Figure 2E**). Combining the results of univariate (**Figure 2F**) and multivariate (**Figure 2G**) Cox analysis, it appeared that risk scores could be an independent prognostic factor compared to other clinical traits.

### Expression profiles and prognostic potency of nine hub genes

**Figure 3:**
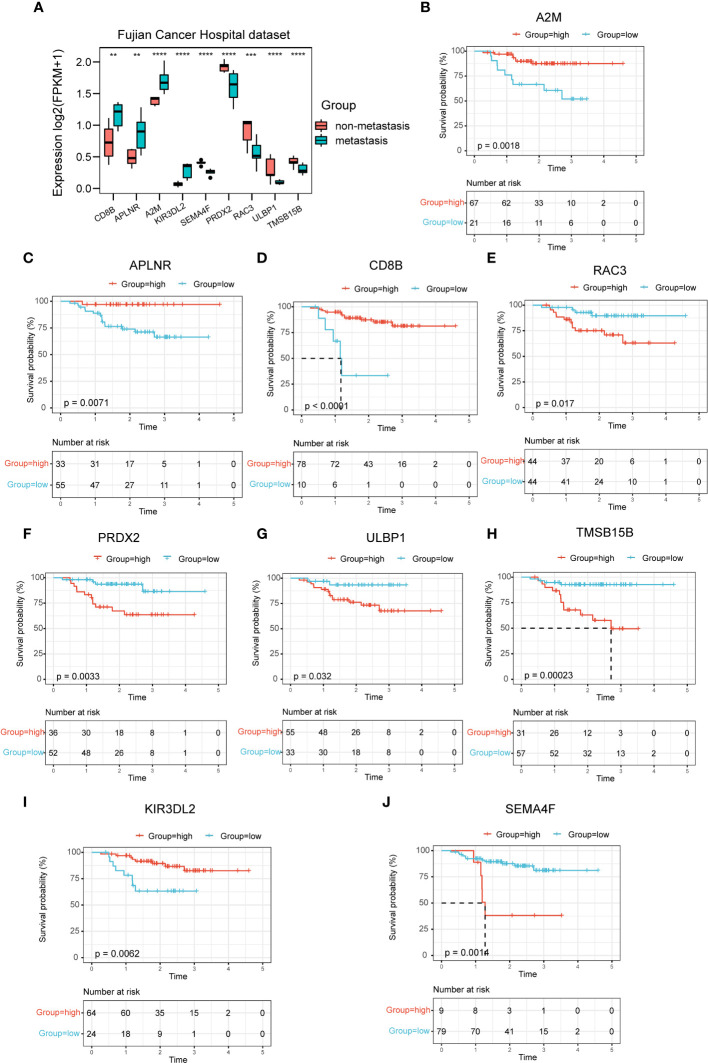
Expression profiles and prognostic potency of nine hub genes. **(A)** The 9 immune-related signatures were significantly different between the non-metastasis group and metastasis group of this hospital cohort. **(B–J)** A2M, APLNR, CD8B, RAC3, PRDX2, ULBP1, TMSB15B, KIR3DL2 and SEMA4F had extraordinary differences of survival probability between the high-risk and the low-risk groups in GSE102349. **P < 0.01, ***P < 0.001, ****P < 0.0001.

In the mRNA sequencing data of NPC from Fujian Cancer Hospital, the expressions of the nine immune-related hub genes were apparently different in the metastasis and non-metastasis groups (**Figure 3A**). Of the 9 genes, CD8B, APLNR, A2M and KIR3DL2 were upregulated in the non-metastasis group where the patients would have lower risk and gain better outcomes. In contrast, SEMA4F, PRDX2, RAC3, ULBP1 and TMSB15B were upregulated in the metastasis group where the patients would have higher risk and suffer worse outcomes (**Figure 3A**). To verify the predictive validity of nine hub genes for prognostic outcome, survival analysis demonstrated promising prognostic differentiation (**Figures 3B–J**).

### Enrichment pathways of hub genes and correlation with oncogenic pathways, proliferative activity

**Figure 4:**
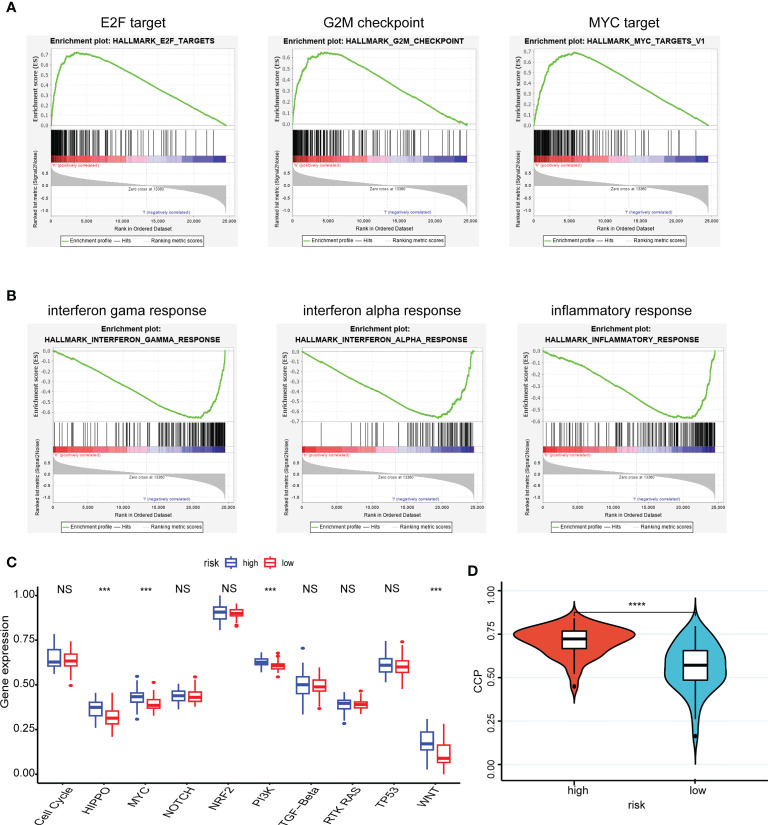
Enrichment pathways of hub genes and correlation with oncogenic pathways, proliferative activity. **(A, B)** The high-risk group **(A)** was mainly distributed in the E2F target, G2M checkpoint and MYC target using the GSEA method and the low-risk group **(B)** was mainly distributed in the INF-γ, INF-α and inflammatory responses using the GSEA method; **(C)** Patient samples from high- and low-risk groups showed significant differences in scores across the ten carcinogenic pathways; **(D)** Patients in the high-risk group having high CCP scores. ***P < 0.001, **** P < 0.0001, ^ns^P > 0.05.

The pathway in which the gene is enriched tends to indicate that the gene plays a role in that physiological process. Using the GSEA method, the high-risk group was mainly distributed into the E2F, G2M checkpoint, and MYC targets, which were closely related to interactions on angiogenesis, extracellular matrix remodeling, and tumor cell-endothelial cell interactions (**Figure 4A**). Correspondingly, the low-risk group was mainly distributed in the INF-γ, INF-α, and inflammatory responses, which were closely related to antitumor effect in anti-tumor immune response (**Figure 4B**).

Moreover, patient samples from high- and low-risk groups showed significant differences in scores across the ten carcinogenic pathways (**Figure 4C**). Patients in the high-risk group had higher oncogenic pathogenic activity, predicting that a higher risk of cancer progression was involved. And the CCP scores corroborated this finding, with patients in the high-risk group having high CCP scores, which suggested that the tumors had stronger proliferative activity (**Figure 4D**).

### Assessment of the tumor immune microenvironment and immune checkpoints

**Figure 5:**
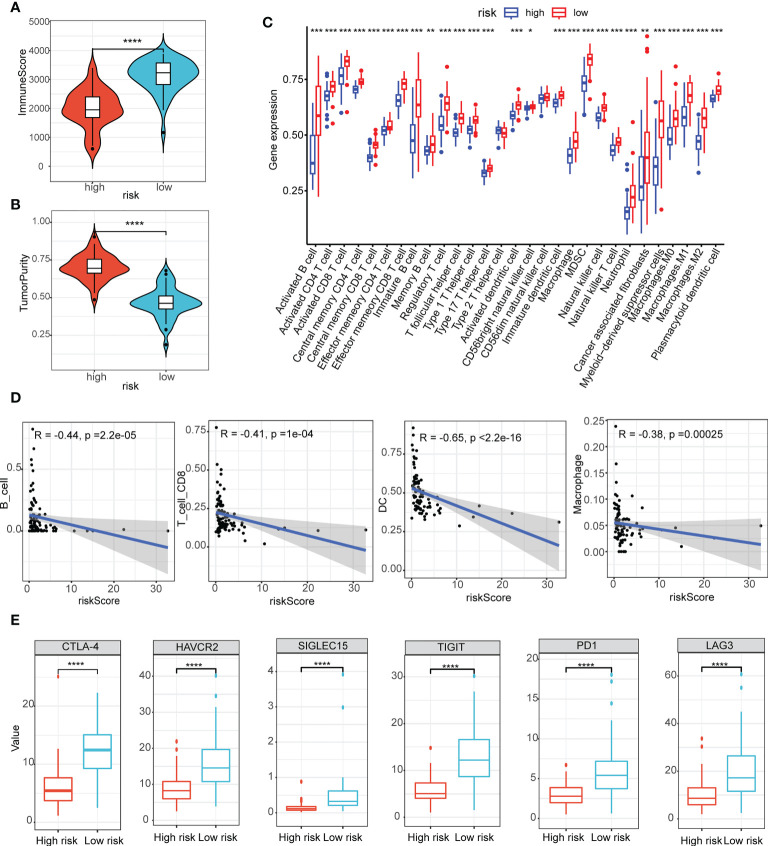
Assessment of the tumor immune microenvironment and immune checkpoints. **(A, B)** The immune scores **(A)** and scores of tumor purity **(B)** between the high- and low-risk group had notable statistical differences in the violin plot; **(C)** The compositions of the 29 immune-cell types were significantly different in the high- and low-risk groups; **(D)** B cells, CD8 T cells, dendritic cells (DC), macrophages infiltration were negatively related to the risk scores; **(E)** Immune checkpoint inhibitors (CTLA-4, HAVCR2, SIGLEC15, TIGIT, PD1 and LAG3) between the high- and low-risk groups had notable statistical differences in the box plots. *P < 0.05, **P < 0.01, ***P < 0.001, ****P < 0.0001.

Here, we estimated how the immune microenvironment differed between patients in high- and low-risk groups in terms of immune scores and levels of immune cell infiltration. The patients from the low-risk group had higher immune scores but lower tumor purity (**Figures 5A, B**). Additionally, the compositions of the 29 immune-cell types were significantly different in the high- and low-risk groups (**Figure 5C**). In the low-risk group, almost all levels of immune cell infiltration were higher than in the high one, including B cells, CD8 T cells, dendritic cells (DC), macrophages (**Figure 5D**). Moreover, there were significant statistical differences in the immune checkpoint inhibitors (CTLA-4, HAVCR2, SIGLEC15, TIGIT, PD1 and LAG3) between the high- and low-risk groups (**Figure 5E**).

### Predictive power for immunotherapy efficacy

**Figure 6:**
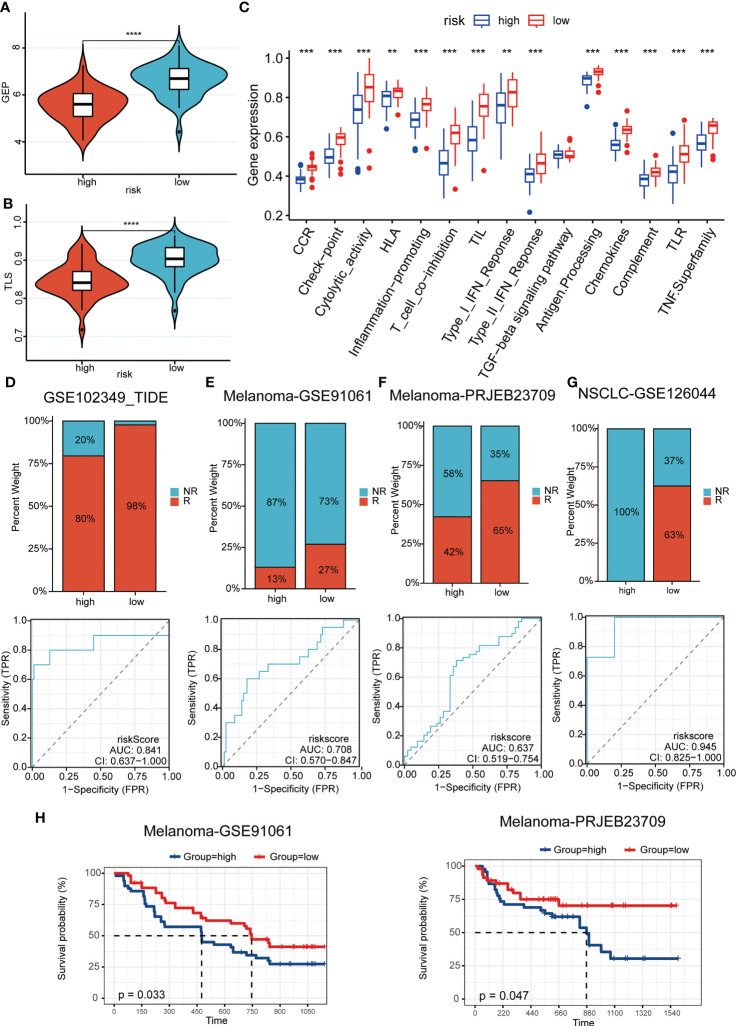
Predictive power for immunotherapy efficacy. **(A, B)** GEP **(A)** and TLS **(B)** score were higher in the low-risk group; **(C)** numerous immune-related indicators were over-expressed in low-risk patients; **(D–G)** Patients in the low-risk group had a higher immune response in a cohort of patients with whether nasopharyngeal carcinoma **(D)** or melanoma **(E-F)**, or non-small cell lung cancer **(G)**; **(H)** The high-risk patients had a worse prognosis when compared with the patients of the low-risk group in melanoma cohorts. **P < 0.01, ***P < 0.001, ****P < 0.0001.

We were the first to evaluate GEP and TLS score, and showed that there were higher levels of immune cell receptors in low-risk patients (**Figures 6A, B**). Subsequently, the same results were observed in numerous immune-related indicators (**Figure 6C**). These results suggested that tumors stimulate more immune cell activation and strong ligand-receptor activation in patients in the low-risk group, laying the biological foundation for a positive response to this immunotherapy. As **Figure 6D–G** showed, patients in the low-risk group had a higher immune response in a cohort of patients with whether nasopharyngeal carcinoma or melanoma, or non-small cell lung cancer. It was evident that the patients of the high-risk group had less chance of benefiting from immunotherapy, which represented a worse prognosis when compared with the patients of the low-risk group (**Figure 6H**).

### Immune landscapes and cellular communication at the single-cell level

**Figure 7:**
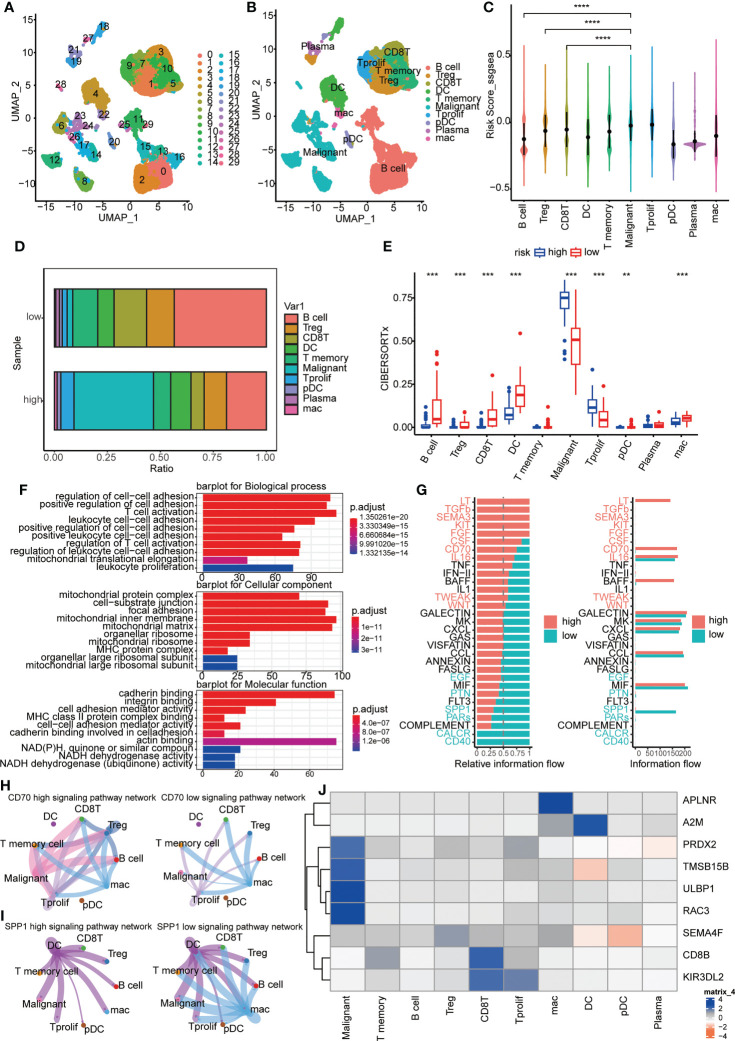
Immune landscapes and cellular communication at the single-cell level. **(A, B)** A cluster of 29 distinct cell types in GSE150430 cohort was defined by two-dimensional spatial visualization of UMAP analysis; **(C)** Risk scores for samples in different cell subsets; **(D)** The proportion of cell composition in high- and low-risk groups; **(E)** Immune cell infiltration in high- and low-risk groups using CIBERSORTx; **(F)** The major pathways enriched for differential genes between high- and low-risk groups; **(G)** Active pathways were observed to vary in the high- and low-risk groups; **(H, I)** CD70 and SPP1 signaling pathways in high- and low-risk groups; **(J)** The intracellular expression of nine hub genes. **P < 0.01, ***P < 0.001, ****P < 0.0001.

A cluster of 29 distinct cell types in GSE150430 cohort was defined by two-dimensional spatial visualization of UMAP analysis (**Figure 7A**). Cell lineages were distributed to each cluster by gene expression with reference to the human primary cell atlas data in TISCH. As a result, cells were annotated (**Figure 7B**). We targeted the most significantly differentially expressed genes in each cluster to better understand the species of cell fascicles (**Figure S3A**). In the identified cell subsets, the GSVA and ssGSEA algorithm was employed to calculate the performance of the nine hub genes at the single-cell level. Significantly higher risk scores were observed in malignant cells than in B cells and CD8 T cells (**Figures 7C**, **S3B**). The same conclusion can be drawn in the cellular localization map (**Figures S3C, D**). Moreover, the percentage of B cells and CD8 T cells in the low-risk samples was notablely higher than that of the high ones; however, the percentage of malignant cells in the high-risk samples was significantly higher than that of the low ones (**Figures 7D, E**). This was consistent with previous findings indicating that the high-risk scores predicted worse biological behavior.

Next, we carried out functional exploration. The major pathways enriched for differential genes between high- and low-risk groups were those related to intercellular adhesion and immune cell activation, suggesting that the response to distant metastasis and immune resistance differed between high and low-risk groups (**Figure 7F**). Also, active pathways were observed to vary in the high- and low-risk groups, like LT, TGFb, SEMA3, KIT, FGF and CD70 pathways being active in the high group while CALCR, CD40, and SPP1 pathways being vibrant in the low group (**Figures 7G**, **S3E**). In **Figures 7H, I**, the distinction of CD70 and SPP1 signaling pathways in high- and low-risk groups was more intuitive. Finally, the intracellular expression of nine hub genes is exhibited (**Figure 7J**). It can be seen that the expression of PRDX2, TMSB15B, ULBP1, and RAC3 was specifically increased in malignant cells, and the high expression of these genes coincides with a worse survival prognosis (**Figures 3E–H**).

## Discussion

In this study, we screened nine hub genes to construct an immune-related risk model from differently expressed genes of metastatic and non-metastatic NPC patients in Fujian Cancer Hospital. The model accurately predicted overall survival and was strongly associated with immune infiltration at both the transcriptome level and the single-cell level.

In NPC, polygenic models for predicting prognosis based on gene expression levels have been rarely reported. More attention has focused on predicting prognosis at the miRNA level, single gene level. Prediction models are constructed by integrating various different factors, such as clinicopathological features, imaging features, genomic features, etc. A study identified a prognostic predictive risk model for patients with nasopharyngeal carcinoma based on three miRNA signatures (ebv-miR-BART19-3p, hsa-miR-135b, hsa-miR-141), which can be used to predict the overall survival of patients with nasopharyngeal carcinoma. (3-year ROC = 0.76) (39). In a CT-based and PET-based signatures for individual induction chemotherapy (IC) in advanced NPC, the researchers proposed a radiomics nomogram with a C-index of 0.754 [95% confidence interval (95% CI), 0.709-0.800] in the training set and 0.722 (95% CI, 0.652-0.792) in the test set (40). Another study investigated the prognostic significance of tumor-infiltrating immune cells and microenvironment-relevant genes in NPC (NPC) and their correlations. A risk score model composed of DARC, IL33, IGHG1, and SLC6A8 was established with a good performance for PFS prediction (AUC = 0.738) (41). In our study, one of the novelties is the construction of a predictive model for metastatic NPC, and the good predictive accuracy achieved. The area under the ROC curve (AUC) of our model was 0.79 at 1-year, and 0.81 at 3-years, respectively, indicating a high predictive value. We filled the research gap of genetic prognostic prediction model for metastatic NPC. The results of the study are expected to provide a theoretical basis for accurate prognostic assessment of metastatic NPC.

Although improving the responsiveness of immunotherapy is very promising for the treatment of metastatic tumors, the effectiveness of strategies to improve the immune response to cancer varies from patient to patient, due to the heterogeneity of cancer cells and immune cells in TME, the crosstalk of biological signaling pathways, and the varying composition of specific immune cells (42). Our study proposes a robust risk prediction model based on metastatic NPC cases in Fujian Cancer Hospital, which can accurately predict the prognosis and immunotherapy efficacy of metastatic NPC patients.

Tumor-infiltrating lymphocytes determine the progression and aggressiveness of tumors and are a source of important prognostic information for patients (43, 44). In this study, samples from the low-risk group had higher immune scores, lower scores of tumor purity, and higher value of immune checkpoint inhibitors simultaneously. It can be reasonably speculated that the patients from the low-risk group will benefit from immunotherapy as compared with patients from the high-risk group. The well-established prognostic model could make an obvious distinction of the patients with metastatic NPC to predict the risk of poor prognosis. For the advanced patients assigned to the low-risk group, the combination of chemoradiotherapy and immunotherapy would be an appropriate choice to attempt a better outcome.

The pro-oncogenic pathways, including E2F, G2M checkpoint and MYC targets pathway, favor tumor cells to promote growth, migration, invasion, and angiogenesis. In our analysis, GESA identified the enrichment of E2F, G2M checkpoint, and MYC targets pathway in the high-risk group, which may contribute to the dismal prognosis. On the contrary, inflammatory response contributes to cancer cell death by inducing an anti-tumor immune response and therefore accounts for a favor prognosis of low-risk group.

Recently, the prediction and evaluation of the efficacy and outcome after immunotherapy for a specific tumor or the patient with a specific tumor is a hot spot in the development of contemporary medical treatment. Tumors and their microenvironments constantly interact with each other (45). According to the type and number of infiltrated immune cells, it can be divided into hot tumors and cold tumors. Hot tumors refer to tumors that have triggered the body’s immune responses with a certain number of immune cell infiltration, which tend to respond well to immune checkpoint inhibitors. While cold tumors are considered as those with few immune cells where it is difficult to stimulate the autoimmune responses and where immune checkpoint inhibitors could not play an effective role when compared with hot tumors. In this study, the risk model we constructed can predict immune cell infiltration in patients and even infer specific immune cell content levels in both transcriptome level and single-cell level. In addition, patients in the high-risk group had a large proportion of malignant cells in their cellular composition, whereas immune cells in the low-risk group had a large proportion. There was also a dramatic difference in the ligand receptors for cellular communication between the high- and low-risk patients. The low-risk group or the non-metastasis group had high immune scores and abundant immune cell infiltration, which means that they have a hot tumor component and superior immune response in their bodies, indicating a higher likelihood of benefiting from immunotherapy and a better prognosis. Therefore, accurate prediction of our model holds great value for individualized treatment and efficacy detection in clinical settings for advanced NPC patients.

Immunotherapy drugs targeting PD-L1 and CTLA-4 are playing an increasingly critical role in the treatment of malignant tumors (46). The expression levels of PD-L1 or other immune checkpoints will directly affect the therapeutic effect of immune checkpoint inhibitors, by which the application of immune checkpoint inhibitors can be guided. TLS is an ectopic lymphoid-like structure that is mostly formed in tissues where inflammation occurs (47). In recent years, many studies have revealed that tumor-infiltrating B lymphocytes (48) and tumor-associated TLS have a non-negligible correlation with the response to immune-checkpoint blockade treatment, which provides a new biological marker for the clinical decision-making of immunotherapy. In this study, there are a higher number of B memory lymphocytes and increased immune checkpoint expression in the low-risk group, which insinuates more opportunity to benefit from immunotherapy. The accuracy of the risk model predictions was likewise validated in multiple immunotherapy cohorts.

To the best of our knowledge, our study presented the first immunopredictive risk model for metastatic NPC based on realistic cases. However, our study had some limitations. A major limitation was the lack of a prospective NPC cohort to validate the prognostic role and stratification performance of the model. In addition, the role of predicting immunotherapy efficacy in real-world settings needs further investigation.

## Conclusions

We have constructed a model based on immunity of metastatic NPC and determined its prognostic value. In addition, the model identified cell-cell communication between tumor and immunity, along with potential therapeutic approaches to target metastatic NPC.

## Data availability statement

The datasets presented in this study can be found in online repositories. The names of the repository/repositories and accession number(s) can be found in the article/**Supplementary Material**.

## Ethics statement

All included patients gave their written informed consent. The study was approved by the Ethics Committee of Fujian Cancer Hospital and Fujian Medical University Cancer Hospital (approval number SQ2019-035-01). The patients/participants provided their written informed consent to participate in this study.

## Author contributions

XCC and QD designed the article. QD, TL and YS organized the public data and wrote the manuscript. XC, TL, WH and XC took charge for data visualization. YS, ZH and YL obtained the clinical information. DW and SQ contributed to the concept and revised the article. All authors contributed to the article and approved the submitted version.

## Supplementary material

Supplementary Figure 1 The schematic diagram of this study.

Supplementary Figure 2
**(A)** PCA cluster analysis of the metastatic group (group M) and the non-metastatic group (group N); **(B, C)** The risk score was significantly distinguished between **(B)** clinical stage I–III and stage IV in GSE102349 (n=73) as well as **(C)** metastatic and the non-metastatic patients in Fujian Cancer Hospital dataset (n=10).

Supplementary Figure 3
**(A)** The cell annotation and the specially expressed genes in each cluster; **(B)** Risk scores for samples in different cell subsets; **(C, D)** Comparison of cellular composition of high and low risk groups; **(E)** The signaling pathway of high and low risk groups in comparison.
