# Supplementary figures and images for: An immune-related prognostic model predicts neoplasm-immunity interactions for metastatic nasopharyngeal carcinoma
Source: Front Immunol. 2023 Mar 31;14:1109503. doi: 10.3389/fimmu.2023.1109503 (PMC10102363; doi:10.3389/fimmu.2023.1109503)

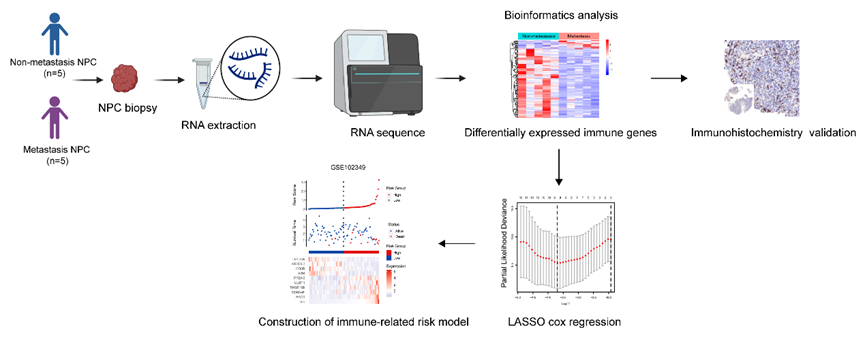

Supplement: Supplementary Figure 1 — The schematic diagram of this study. [file Image_1.tif]

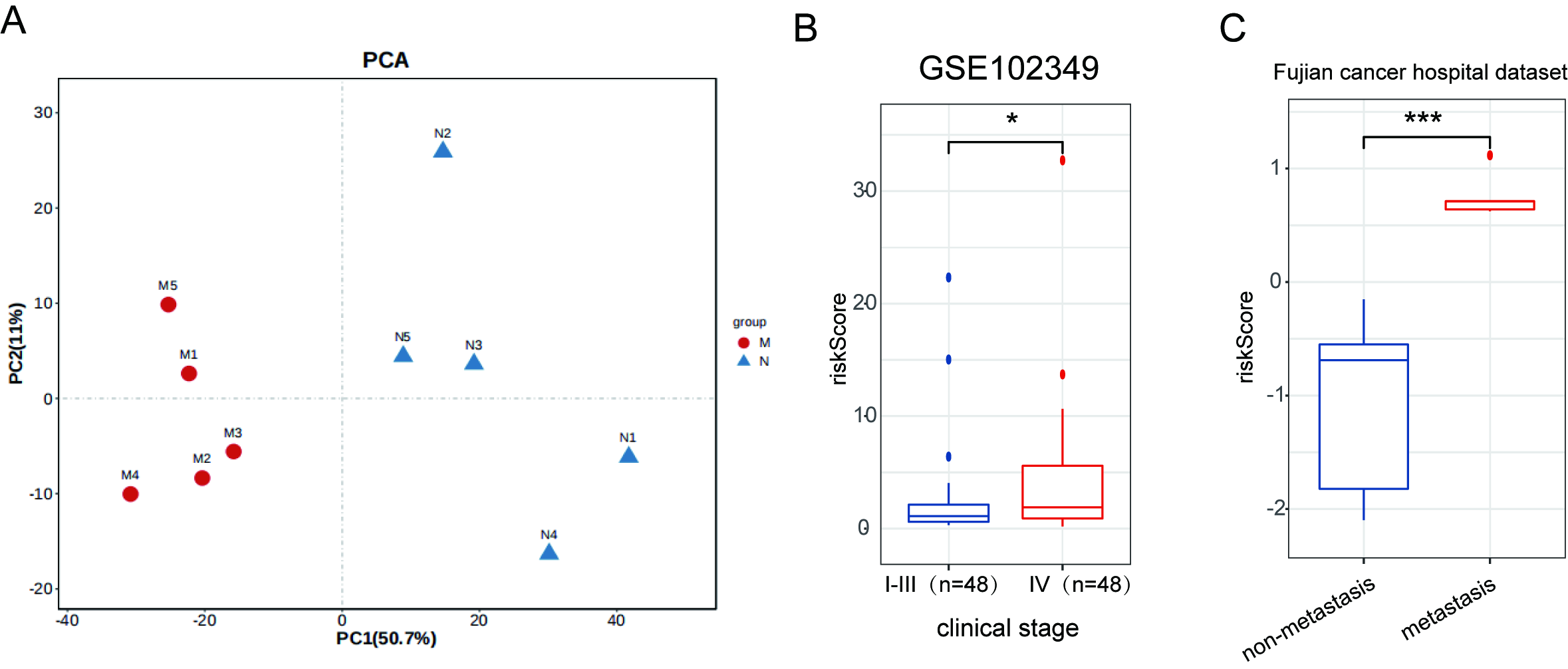

Supplement: Supplementary Figure 2 — (A) PCA cluster analysis of the metastatic group (group M) and the non-metastatic group (group N); (B, C) The risk score was significantly distinguished between (B) clinical stage I–III and stage IV in GSE102349 (n=73) as well as (C) metastatic and the non-metastatic patients in Fujian Cancer Hospital dataset (n=10). [file Image_2.tif]

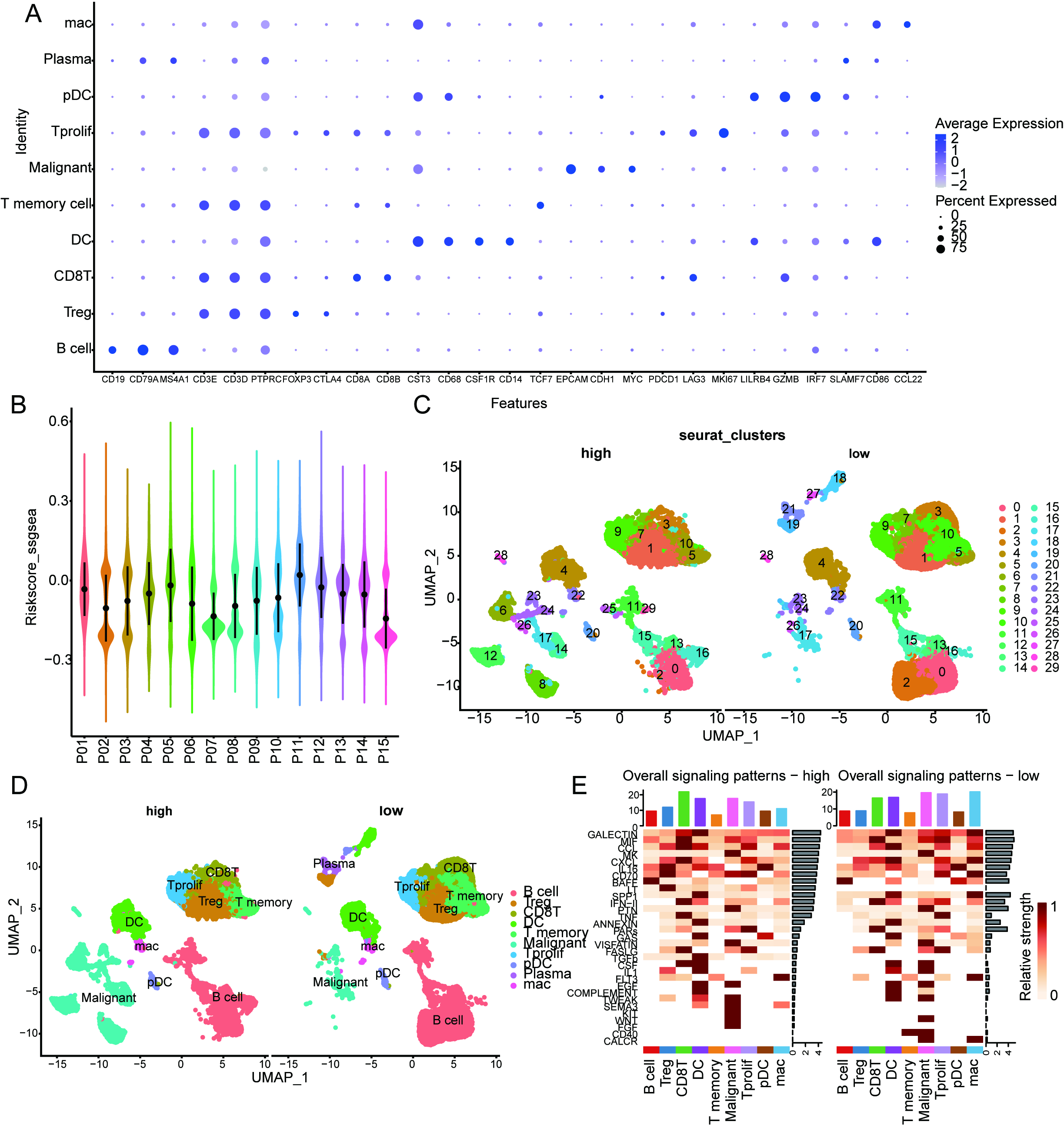

Supplement: Supplementary Figure 3 — (A) The cell annotation and the specially expressed genes in each cluster; (B) Risk scores for samples in different cell subsets; (C, D) Comparison of cellular composition of high and low risk groups; (E) The signaling pathway of high and low risk groups in comparison. [file Image_3.tif]
